# *Lactobacillus reuteri* V3401 Reduces Inflammatory Biomarkers and Modifies the Gastrointestinal Microbiome in Adults with Metabolic Syndrome: The PROSIR Study

**DOI:** 10.3390/nu11081761

**Published:** 2019-07-31

**Authors:** Carmen Tenorio-Jiménez, María José Martínez-Ramírez, Isabel Del Castillo-Codes, Carmen Arraiza-Irigoyen, Mercedes Tercero-Lozano, José Camacho, Natalia Chueca, Federico García, Josune Olza, Julio Plaza-Díaz, Luis Fontana, Mónica Olivares, Ángel Gil, Carolina Gómez-Llorente

**Affiliations:** 1 Endocrinology and Nutrition Clinical Management Unit, University Hospital Virgen de las Nieves, 18014 Granada, Spain; 2 Endocrinology and Nutrition Clinical Management Unit, University Hospital of Jaén, 23007 Jaén, Spain; 3 Department of Health Sciences, School of Health Sciences, University of Jaén, 23071 Jaén, Spain; 4 Digestive Diseases Clinical Management Unit, University Hospital of Jaén, 23007 Jaén, Spain; 5 Department of Signal Theory, Networking, and Communications, University of Granada, 18071 Granada, Spain; 6 Department of Microbiology, University Hospital Campus de la Salud, 18016 Granada, Spain; 7 Instituto de Investigación Biosanitaria ibs. GRANADA, 18012 Granada, Spain; 8 Department of Biochemistry and Molecular Biology II, School of Pharmacy, University of Granada, 18071 Granada, Spain; 9 Institute of Nutrition and Food Technology “José Mataix”, Center of Biomedical Research, University of Granada, 18016 Granada, Spain; 10 CIBEROBN (CIBER Physiopathology of Obesity and Nutrition), Instituto de Salud Carlos III, 28029 Madrid, Spain; 11 Biosearch Life, 18004 Granada, Spain

**Keywords:** metabolic syndrome, gastrointestinal microbiome, *Lactobacillus reuteri* V3401, probiotics, obesity

## Abstract

Previous studies have reported that probiotics may improve clinical and inflammatory parameters in patients with obesity and metabolic syndrome (MetS). *Lactobacillus (L.) reuteri* V3401 has shown promising results on the components of MetS in animal studies. We aimed to evaluate the effects of *L. reuteri* V3401 together with healthy lifestyle recommendations on adult patients with MetS. Methods: We carried out a randomized, crossover, placebo-controlled, single-center trial in which we included 53 adult patients newly diagnosed with MetS. Patients were block randomly allocated by body mass index (BMI) and sex to receive a capsule containing either the probiotic *L. reuteri* V3401 (5 × 10^9^ colony-forming units) or a placebo once daily for 12 weeks. Anthropometric variables, biochemical and inflammatory biomarkers, as well as the gastrointestinal microbiome composition were determined. Results: There were no differences between groups in the clinical characteristics of MetS. However, we found that interleukin-6 (IL-6) and soluble vascular cell adhesion molecule 1 (sVCAM-1) diminished by effect of the treatment with *L. reuteri* V3401. Analysis of the gastrointestinal microbiome revealed a rise in the proportion of *Verrucomicrobia*. Conclusions: Consumption of *L. reuteri* V3401 improved selected inflammatory parameters and modified the gastrointestinal microbiome. Further studies are needed to ascertain additional beneficial effects of other probiotic strains in MetS as well as the mechanisms by which such effects are exerted.

## 1. Introduction

Obesity is a chronic disease, affecting developed and developing countries, that has multiple comorbidities and deteriorates quality of life. It is characterized by an increase of fat mass, which can consequently produce hypertrophy of the adipocytes, leading to an altered adipose tissue functionality. Individuals who are obese can develop an insulin resistance syndrome, also called metabolic syndrome (MetS). MetS is defined by insulin resistance, dyslipidemia, hypertension, and increased abdominal circumference, and it is associated with the development of type 2 diabetes (DM2), cardiovascular disease (CVD), and nonalcoholic fatty liver disease (NAFLD). This condition is associated with a two-fold increase in the risk of coronary heart disease, cerebrovascular disease, and a 1.5-fold increase in the risk of all-cause mortality [1], constituting a major public health challenge worldwide.

Nowadays, there is sound evidence linking the metabolic dysfunction seen in MetS to a proinflammatory state. Adipose tissue is, in part, responsible of this low-grade inflammatory state through the increasing release of proinflammatory molecules, such as leptin and tumor necrosis factor α (TNF-α), and the inhibition of adiponectin secretion, an anti-inflammatory adipokine [2]. In recent years, it has become evident that alteration of the gastrointestinal microbiome, also called gastrointestinal dysbiosis, may also contribute to the development of insulin resistance associated with obesity [3,4,5]. Furthermore, different studies have linked gastrointestinal dysbiosis with the development of obesity and other hallmarks of MetS [6,7]. In this sense, a decreased ratio of *Bacteroidetes/Firmicutes* has been described in individuals who are obese compared to normal-weight individuals [7]. Likewise, individuals with a low bacterial richness have more dyslipidemia, insulin resistance, inflammatory phenotype, and overall adiposity than individuals with high bacterial richness [8]. In addition, an aberrant gastrointestinal microbiome can promote subacute systemic inflammation, insulin resistance, and increased risk of CVD by mechanisms that include exposure to bacterial products, such as lipopolysaccharide (LPS), which is responsible for the metabolic endotoxemia related to MetS [9].

In the last years, treatment of the hallmarks of MetS with probiotics has emerged as a promising therapy. Probiotics are living microorganisms that confer health benefits to the host when administered in adequate amounts [10]. *Bifidobacterium* and *Lactobacillus* are the most frequently used genera of probiotics used in humans. Some of the beneficial effects of probiotics are mediated by their capacity to normalize the gastrointestinal microbiome, reinforce the gut barrier function composition [11,12], and their immunomodulatory actions [12,13]. Therefore, the addition of probiotics to a healthy diet could represent an interesting tool to fight obesity, MetS, and associated inflammation when used alongside dietary management and lifestyle modifications (e.g., increased physical activity). In this regard, some studies have found an improvement of anthropometric parameters and a decrease in inflammatory biomarkers in this disease after probiotic administration [14]. However, the beneficial effects of probiotics on MetS components are contradictory [15], probably because of the different probiotic strains, doses, and clinical study designs.

*Lactobacillus (L.) reuteri* V3401 strain, deposited in the Spanish Type Culture Collection (CECT) with accession number CECT 8695, was isolated from cow’s raw milk on Mark, Rogosa and Sharper (MRS) agar medium, and 16S gene sequence analysis was carried out for its identification. In addition, its carbohydrate fermentation ability was characterized by the Analytical Profile Index (API) CH50 test [16]. This strain has been shown to reduce the absorption of fluoresterol, a fluorescent cholesterol analogue, by HT-29 human enterocytes [16]. Furthermore, Wistar rats fed a hypercholesterolemic diet supplemented with the probiotic strain for 57 days showed HDL levels similar to those of a healthy control group fed a standard diet [16]. Regarding glycemic levels, hypercholesterolemic animals supplemented with the probiotic strain showed similar values to those of normocholesterolemic mice, whereas animals under a hypercholesterolemic diet without the probiotic strain exhibited higher levels than normocholesterolemic mice [16]. Higher glucose levels are related to insulin resistance, which is normally associated with hypercholesterolemia and low HDL levels, both of them components of MetS. In this setting, supplementation with *L. reuteri* V3401 might offer an additional metabolic advantage together with healthy diet and exercise recommendations in patients with MetS.

All things considered, the present study aimed to evaluate whether the consumption of the probiotic strain *L. reuteri* V3401, together with healthy lifestyle (hypocaloric diet and physical activity) recommendations, was capable of improving MetS components. For this purpose, we designed a double-blind, crossover, placebo-controlled, single-center, randomized clinical trial (RCT).

## 2. Materials and Methods

### 2.1. Ethical Statement

All research and procedures performed during the study complied with the Declaration of Helsinki and the Guidelines of Good Clinical Practice. After receiving a complete verbal description of the study, patients signed a written informed consent. The study protocol was approved by the local Ethics Committee of both Granada and Jaén (references CEI-Jaén 25022016 and CEI-Granada 28022016, respectively).

### 2.2. Subjects and Experimental Design

We performed a randomized, double-blind, crossover, placebo-controlled, single-center trial in patients with a new diagnosis of MetS, according to the criteria of the International Diabetes Federation (IDF). The complete study design including sample size, randomization, and the trial protocol have been previously published [17] and registered at www.clinicaltrials.gov as NCT02972567. The study was conducted in agreement with the Standard Protocol Items: Recommendations for Interventional Trials (SPIRIT) guidelines.

Sample size was calculated based on the range and median value of lipopolysaccharide (LPS) and assuming a power of 80% and a significant level of 5% [17]. In brief, a total of 53 out of 60 adult patients were recruited at the Endocrinology and Nutrition Clinical Management Unit, University Hospital of Jaén (Jaén, Spain) by qualified personnel. Patients were block randomly allocated, by BMI and sex, in a 1:1 ratio to receive a capsule containing either the probiotic *L. reuteri* V3401 (5 × 10^9^ colony-forming units) or the placebo (maltodextrin) once daily for 12 weeks.

**Figure 1:**
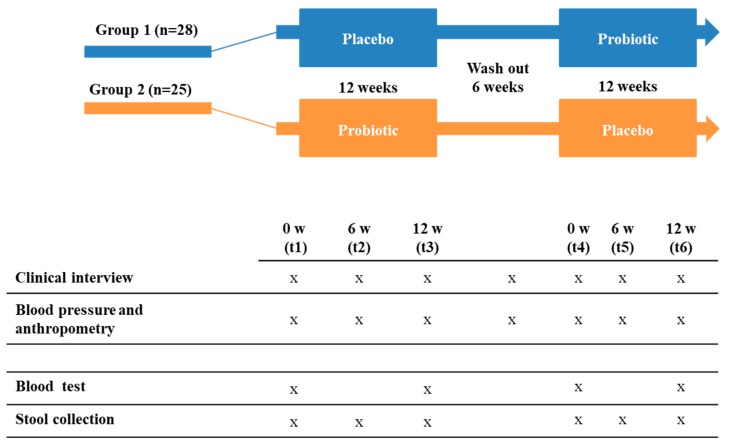
Illustration of the experimental design: A randomized, double-blind, crossover, placebo-controlled, single-center trial comparing the effect of consumption of *Lactobacillus reuteri* V3401 for 12 weeks on various clinical, biochemical, and inflammatory biomarkers and gastrointestinal microbiota. w: weeks; t: time; x: sample collection.

Both capsules, probiotic and placebo, were provided by Biosearch Life (Granada, Spain). In addition, participants received an intensive lifestyle intervention program that included nutritional and physical counseling to achieve and maintain a 7% loss of initial body weight and increase moderate-intensity physical activity for at least 150 min/week. In Figure 1 we summarized the experimental design of the study.

### 2.3. Anthropometric, Biochemical, Inflammatory, and Cardiovascular Data

We performed a systematic symptom evaluation on each visit, with special emphasis on gastrointestinal symptoms, and a physical examination. Body weight (kg), height (cm), and waist circumference (cm) were measured by the same person using standardized procedures. Blood pressure was taken 3 times by the same person, and the mean of the three values was included. The biochemical analyses, including lipid and glucose metabolism, were performed at the University Hospital of Jaén following internationally accepted quality control protocols. Homeostasis assessment model for insulin resistance (HOMA-IR) was calculated using fasting plasma glucose and insulin values.

Blood samples were collected from each patient and after 12 h of fasting, at the beginning and the end of each intervention period. Serum and plasma samples were collected by centrifugation of blood samples and kept at −80 °C until analysis.

Plasma adipokines as well as cardiovascular and inflammatory biomarkers—adiponectin, leptin, resistin, IL-6, IL-8, TNF-α, total plasminogen activator inhibitor-1 (PAI-1), hepatocyte growth factor (HGF), monocyte chemoattractant protein 1 (MCP-1), soluble intracellular adhesion molecule 1 (sICAM-1), soluble vascular cell adhesion molecule 1 (sVCAM-1), and myeloperoxidase (MPO)—were analyzed on a Luminex 200 system (Luminex Corporation, Austin, TX, USA) with human monoclonal antibodies (EMD Millipore Corp, Billerica, MA, USA) using MILLIplex™ kits (HADK1MAG-16K, HSTCMAG-28SK, HAD2MAG-61K, HCVD2MAG-67K) according to the manufacturer’s recommendations.

LPS and LPS-binding protein (LBP) were determined in serum samples using CEB526GE and SEB406 HU ELISA kits (Cloud-Clone Corp, TX, USA), respectively, following the manufacturer’s instructions.

### 2.4. Fecal Samples, DNA Extraction, and Next-Generation Sequencing

Fecal samples were collected from each patient at each time (t1, t2, t2, t4, t5, and t6). Fecal samples were placed inside of a sterile plastic bottle and kept at −80 °C until analysis. DNA was extracted using a QIAamp DNA stool Mini Kit (QIAGEN, Barcelona, Spain) according to the manufacturer’s instructions, with the exception that samples were incubated with the lysis buffer at 95 °C instead of 70 °C to guarantee the lysis of both Gram-positive and Gram-negative bacteria. Extracted DNA samples were sequenced at facilities of the Department of Microbiology, University Hospital Campus de la Salud (Granada, Spain). A 16S metagenomics sequencing was performed following the Illumina protocol.

In summary, the V3-V4 region of the bacterial 16S rRNA gene was amplified using the primers described by Klindworth et al., 2013 [18]. The PCR mixture was composed of 5 μL for each forward and reverse primers (1 μM, Macrogen, Seoul, Korea), 2.5 μL of DNA template samples, and 12.5 μL of 1x Hot Master Mix (KAPA HiFi HS RM, Roche, Basilea, Switzerland) to a final volume of 25 μL. Five microliters of elution solution was used for the negative control. The PCR conditions were: initial denaturation at 95 °C for 3 min, followed by 25 cycles of denaturation at 95 °C for 30 s, primer annealing at 55 °C for 30 s, extension at 72 °C for 30 s, and a final elongation at 72 °C for 5 min. The PCR products were demonstrated by electrophoresis on a 2% agarose gel. No amplification product was observed in the negative control. The amplifications were subjected to purification using Ampure beads (Agencourt Bioscience, La Jolla, CA, USA), the eluted DNA product was quantified using the assays of the Qubit kit (Invitrogen, Life Technologies, Waltham, Massachisetts, USA), and then all samples were pooled in equal concentrations for sequencing. Bioanalyzer 2100 was used with the DNA 1000 Chip kit (Agilent, Palo Alto, CA, USA) to evaluate the quality of the final products for each sample individually. Sequencing was carried out using Illumina MiSeq paired-end sequencing in an Illumina MiSeq device (Illumina Inc., San Diego, CA, USA) with 600 cycles (300 cycles for each paired reading and 12 cycles for the sequence of bar codes) according to the manufacturer’s instructions. Sequence analysis was performed using the metagenomic workflow based on 16S of MiSeq Reporter v2.3 (Illumina Inc., San Diego, CA, USA).

### 2.5. Taxonomic Analysis

The “Quantitative Insights Into Microbial Ecology 2” (QUIIME 2) package was used to analyze sequence data [19]. Denoising quality, chimera check, and clustering were performed using the DADA2 plugins implemented in QUIIME 2. Amplicon sequence variants (ASVs) with a relative proportion lower than 0.1% were eliminated; as a result, the total numbers of ASV were reduced to 2015 but with a very low impact on the total data. The GreenGenes database (version 13.8), together with the naïve Bayes algorithm, was used as the reference 16S database.

### 2.6. Statistical Analysis

For the anthropometric, biochemical, and inflammatory biomarkers, results are presented as the mean values ± standard deviation (SD), unless otherwise indicated. For those variables not following a normal distribution, we applied the logarithmic transformation (insulin, HOMA index, glycated hemoglobin, total cholesterol, triacylglycerols, alanine aminotransferase (GPT), gamma glutamiltransferase (γGT), C reactive protein (CRP), IL-6, IL-8, adiponectin, resistin, HGF, sICAM, sVCAM and LBP) or the inverse transformation (high-density lipoprotein (HDL), aspartate aminotransferase (GOT).

Only patients with less than 5 missing data were considered, resulting in a final number of 34 patients. Missing data in these patients were imputed using principal component analysis (PCA) and trimmed score regression (TSR) [20]. The treatment effect in anthropometric, biochemical, and inflammatory biomarkers was evaluated according to the approach described by Wellek et al. [21]. Two tests were carried out: (i) a pretest for significance of carryover effects, and (ii) a test for significance of treatment effects. The treatment effects were considered significant for those biomarkers for which the null hypothesis of the pretest was not rejected and the null hypothesis of the test was rejected (*p* < 0.05), confirming that the biomarker presented statistically significant differences only due to treatment effects. *p*-value computations were confirmed with different state-of-the-art multivariate approaches, including multivariate analysis of variance (MANOVA) [22], partial least-squares discriminant analysis (PLS-DA) [23], and ANOVA simultaneous component analysis (ASCA) [24]. TheMEDA toolbox (https://github/josecamachop/MEDA-Toolbox) and the MANCOVAN toolbox (http://www.mathworks.com/matlabcentral/fileexchange/27014-mancovan) in Matlab (Mathworks) were used to perform the statistical analysis.

For the gastrointestinal microbiota analysis, the generated sequences, ASV, were normalized by means of the rarefaction method (Figure S1). The alpha diversity was measured by means of the Shannon index, whereas the unique fraction metric (Unifrac), both weighted and unweighted, was used to determine the beta diversity. When comparing the incremental of relative bacteria proportions before and after treatment (delta), a pairwise Wilcoxon signed-rank test was used. *p*-values were adjusted by False discovery rate-FDR (*q*-values). 

## 3. Results

### 3.1. Anthropometric, Biochemical, and Inflammatory Data

Anthropometric and biochemical characteristics of the subjects are described in Table 1, whereas in Table 2 the inflammatory biomarkers determined in blood samples are described.

**Figure 2:**
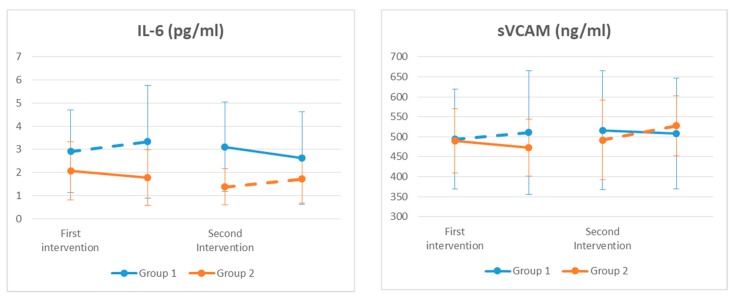
Inflammatory biomarkers throughout the study. The levels of interleukin 6 (IL-6) and soluble vascular cell adhesion molecule 1 (sVCAM) were modified by the probiotic consumption (*p* < 0.05). Continuous line: probiotic group. Discontinuous line: placebo group.

In the case of BMI, diastolic blood pressure, GOT, and LBP, we found that the washout period was not long enough to avoid the carryover effects. We found significant differences for Il-6, sVCAM (Figure 2), and insulin levels (Table 1); however, we did not find any significant results for HOMA index (Table 1).

### 3.2. Gastrointestinal Microbiome Composition

**Figure 3:**
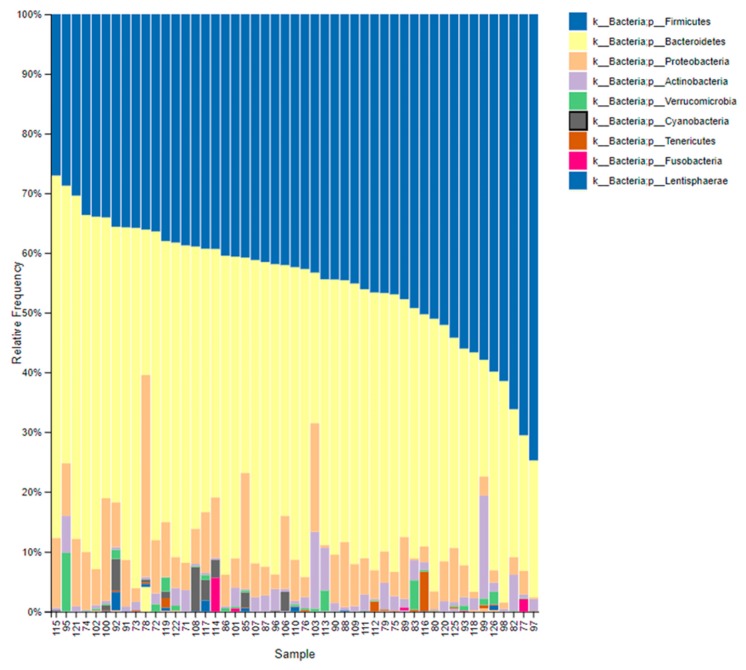
Baseline gastrointestinal microbiome composition. Taxonomic composition of the gastrointestinal communities at the beginning of the intervention. The figure shows bar charts of the relative abundance of bacteria at the phylum level. Each column represents a participant.

We characterized the gastrointestinal microbiome composition of the participants at the beginning, middle, and end of each intervention period (Table S1). However, we were unable to determine the specific presence of the *L. reuteri* V3401 strain in fecal samples due to the lack of specific primers for this strain. As shown in Figure 3, at the beginning of the intervention, the most abundant phyla were *Firmicutes* and *Bacteroidetes* followed by *Proteobacteria*, *Actinobacteria*, *Verrucomicrobia*, and *Cyanobacteria*.

**Figure 4:**
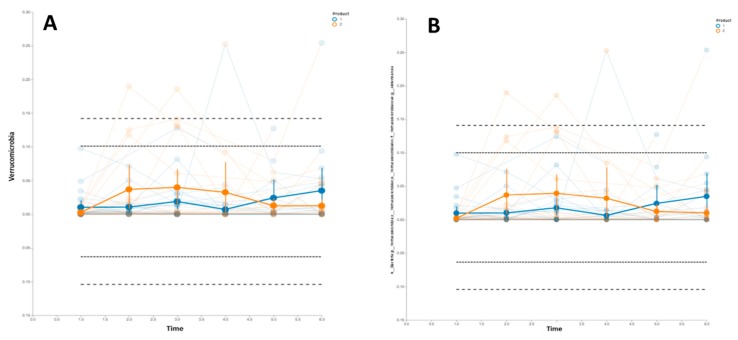
Trend of the relative proportion of *Verrucomicrobia* phylum and *Akkermansia* genus. The thick lines with error bars represent the means of both groups (Group 1: blue lines; group 2: orange lines). Dashed lines represent the means ± 2 and 3 standard deviations. Group 1 started the intervention receiving the placebo (t1, t2, and t3) and then was switched to receive the probiotic strain (t4, t5, and t6). Group 2 started the intervention receiving the probiotic strain (t1, t2, and t3) and then was switched to receive the placebo (t4, t5, and t6). (**A**) Temporal trend of *Verrucomicrobia* phylum; (**B**) Temporal trend of *Akkermansia* genus.

Regarding the bacterial diversity, we did not find significant differences in the alpha diversity throughout the study, measured as the Shannon index (H) (Figure S2), or in the beta diversity (Figure S3). Therefore, our next analysis was to determine the evolution of the relative proportion of specific taxa, namely *Firmicutes*, *Bacteroidetes*, *Verrucomicrobia*, *Actinobacteria*, *Proteobacteria*, *Fusobacteria*, *Cyanobacteria*, *Elusimicrobia*, *Tenericutes*, and *Lentisphaerae*. It is worth mentioning there was an increase in the relative proportion of the *Verrucomicromia* phylum in the participants that consumed the probiotic strain (Figure 4A). The same results were found in the *Akkermansia* genus (Figure 4B).

**Figure 5:**
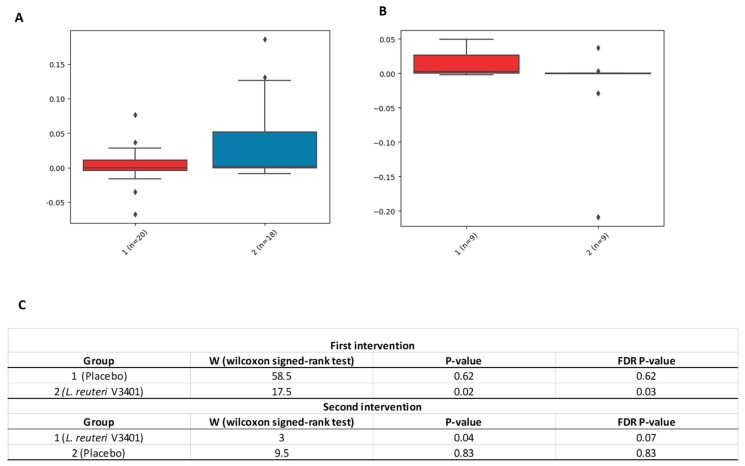
Relative abundance of the delta values of *Verrucomicrobia* phylum. The box plots indicate the relative abundance of *Verrucomicrobia* phylum between the final and the beginning points of each intervention—t3-t1 (**A**) and t6-t4 (**B**)—due to the treatment and the number of patients (n) of each group and treatment. (**A**) shows data for the first part of this intervention study (First intervention, 1: placebo; 2: *L. reuteri* V3401), whereas (**B**) shows data from the intervention after the crossover (Second intervention, 1: *L. reuteri* V3401; 2: placebo). (**C**) describes the Wilcoxon signed-rank test values and the significant levels by means of *p* and FDR *p*-values. FDR: False discovery rate; ♦: outliers data values.

Based on the results described above, we decided to determine whether there were significant differences in the relative abundance of these taxa due to the treatment (probiotic versus placebo). We, therefore, performed a pairwise comparison [25]. During the first intervention (t1, t2, and t3) we observed a significant increase in the delta values (t3–t1) in the *Verrucomicrobia* phylum due to the treatment (probiotic versus placebo). However, during the crossover intervention, the differences (t6–t4) were not statistically significant, although we found a significant trend (FDR *p* = 0.07) (Figure 5).

## 4. Discussion

To the best of our knowledge, the PROSIR study is the first randomized, crossover clinical trial in humans that evaluates whether the strain *L. reuteri* V3401 is capable of improving the components of MetS in humans when added to a healthy lifestyle. We did not find any differences in the clinical features of the syndrome between groups. This may be due to the fact that all subjects included in the study lost weight and improved their metabolic status as a result of the counseling to follow a healthy lifestyle that included diet and physical activity. However, we did find a decrease in IL-6 and sVCAM levels in patients who consumed the probiotic strain, together with a modification of the gastrointestinal microbiome, in particular, an increase in the *Verrucomicrobia* phylum.

Other studies have shown that consumption of *Lactobacillus casei* Shirota reduces sVCAM-1 levels in individuals who suffer from MetS, although in this study no effects on insulin sensitivity, endothelial function, or the inflammatory biomarkers were observed [26]. Bernini et al. [14] showed in another work that consumption of fermented milk enriched with *Bifidobacterium lactis* HN019 resulted in a reduction in BMI, an improvement in the lipid profile, and a significant decrease in proinflammatory cytokines (TNF-α and IL-6).

Systemic low-grade inflammation has an important role in the development of MetS. In this sense, IL-6 is a cytokine that has been associated with insulin resistance. Specifically, IL-6 is able to induce insulin resistance in both liver and adipocytes through reduction of phosphorylation of the insulin receptor substrate (IRS), or by transcription inhibition of the IRS [27,28]. In addition, adhesion molecules, such as sVCAM-1, are necessary for normal development and function of the heart and blood vessels, and they have been related to the development of CVD [29]. In the adult Spaniard population, impaired glucose metabolism has been related to increased levels of sVCAM-1 [30].

Results regarding the utility of probiotics in the treatment of MetS have been contradictory. This may be due to various facts: (i) the particular probiotic strain used in each trial; (ii) the experimental design—most of the studies have been parallel-group randomized trials, whereas a crossover study is a more appropriated approach to determine health benefits of clinical interventions; in crossover studies each participant serves as their own control, but in addition, this clinical design demands a lower sample size than parallel-group studies [21]; and (iii) the duration of the treatment. In our study, 12 weeks might not have been a long enough treatment to reverse, or at least improve, a chronic proinflammatory state as the one observed in MetS.

In recent years, it has become clear that the gut microbiota plays a role in the development of MetS. Specific bacterial groups have been described to be involved in obesity and related metabolic diseases. Among these bacteria, *Akkermansia muciniphila* has been proposed as a contributor to the maintenance of gut health and glucose homeostasis [31]. Administration of *A. muciniphila* to diet-induced obese animals improve their metabolic endotoxemia adipose tissue inflammation and insulin resistance [32]. It is worth mentioning that *Akkermansia* was the only genus of the *Verrucomicrobia* phylum present in the gastrointestinal samples [33]. In humans, *A. muciniphila* has been found to be decreased in prediabetic patients compared to normal glucose tolerance subjects [34]. Conversely, other studies have shown an increase of *A. muciniphila* in type 2 diabetes [35]. More recently, *A. muciniphila* has been reported to be associated with a healthy metabolic status in overweight and obese individuals, in agreement with previous results in murine studies [31]. Additionally, higher *A. muciniphila* abundance has been described in subjects with high bacterial gene richness, which is associated with a healthier metabolic status, in French and Danish population [8,31]. Although we found an increase in the proportion of the *Verrucomicrobia* phylum in the group that received the probiotic, we did not find any significant correlation between the delta proportions of *Verrucomicrobia* and any inflammatory biomarker.

It is important to highlight the age of the subjects that participated in our study. Our patients were younger compared to other previously published trials [14,15,36], and still we observed a decrease of inflammatory biomarkers and an increase in the abundance of *Verrucomicrobia* phylum in this pretreatment phase of the disease. This is consistent in both intervention periods, although in the case of gastrointestinal microbiome, the results only showed a trend in the second intervention, probably because of the dropout number we had, which is usual in large clinical intervention studies.

## 5. Conclusions

In conclusion, our data point to a beneficial effect of supplementation with *L. reuteri* V3401 in subjects with MetS when added to a hypocaloric diet and regular physical activity. In particular, these effects may be mediated by an improvement of dysbiosis and a decreased proinflammatory state, both features of this condition. However, further studies with longer periods of intervention are needed, in animals and clinical studies, to confirm these results and to elucidate the underlying mechanisms of action.

## Figures and Tables

**Table 1:** Anthropometric and biochemical characteristics of the patients.

	Group 1				Group 2			
	Placebo		Probiotic		Probiotic		Placebo	
	t1	t3	t4	t6	t1	t3	t4	t6
Weight (kg)	109.02 ± 26.7	105.70 ± 26.2	101.50 ± 24.5	101.08 ± 24.2	103.49 ± 15.2	96.56 ± 16.2	93.91 ± 16.9	92.0 ± 17.3
BMI (kg/m²)	38.76 ± 7.2	37.57 ± 7.1	36.77 ± 6.8	36.56 ± 6.6	38.30 ± 7.3	35.69 ± 7.1	34.57 ± 6.9	33.80 ± 6.6
SBP (mm Hg)	137.68 ± 16.9	133.28 ± 15.4	133.11 ± 20.4	132.21 ± 14.6	139 ± 22.6	129.95 ± 16.0	131.30 ± 20.0	131.40 ± 18.6
DBP (mm Hg)	84.28 ± 9.6	81.96 ± 7.7	81.68 ± 11.0	82.11 ± 10.5	87.95 ± 14.3	78.18 ±10.4	78.85 ± 12.4	81.60 ± 11.2
Glucose (mg/dL)	103.29 ± 11.0	108.08 ± 11.5	106.74 ± 8.9	105.53 ± 10.5	101.0 ± 13.9	103.68 ± 13.4	101.22 ± 11.8	103.78 ± 16.5
Insulin (mU/mL)	17.50 ± 10.6	16.18 ± 11.3	22.44 ± 10.3	21.74 ± 11.7	14.24 ± 8.5	12.42 ± 10.9	14.04 ± 6.1	17.47 ± 7.8
HOMA index	4.48 ± 2.8	4.41 ± 3.3	5.91 ± 2.8	5.66 ± 3.5	3.71 ± 2.7	3.52 ± 3.6	3.64 ± 1.7	4.46 ± 2.2
Glycated Hemoglobin (%)	5.59 ± 0.4	6.04 ± 2.2	5.44 ± 0.3	5.49 ± 0.3	5.68 ± 0.4	5.90 ± 1.9	5.46 ± 0.3	5.44 ± 0.3
Total cholesterol (mg/dL)	232.42 ± 43.0	207.08 ± 36.0	202.79 ± 45.8	224.16 ± 45.5	233.41 ± 46.5	203.64 ± 37.9	209.56 ± 58.0	220.89 ± 53.8
Triacylglycerols (mg/dL)	119.25 ± 47.6	122.46 ± 59.9	109.00 ± 47.3	118.89 ± 52.2	130.55 ± 47.5	128.23 ± 57.6	112.89 ± 42.7	100.56 ± 62.6
LDL (mg/dL)	156.79 ± 35.7	131.71 ± 30.0	128.11 ± 32.5	144.42 ± 39.0	161.0 ± 41.6	132.91 ± 32.1	136.22 ± 47.7	145.72 ± 44.5
HDL (mg/dL)	50.54 ± 14.6	50.46 ± 12.2	52.47 ± 13.3	54.11 ± 10.2	45.95 ± 9.5	44.68 ± 7.9	50.44 ± 10.3	54.61 ± 11.5
GOT (U/L)	25.75 ± 7.7	22.83 ± 6.3	22.63 ± 6.8	22.32 ± 5.7	23.55 ± 12.4	25.50 ± 14.9	20.56 ± 6.1	21.33 ± 6.2
GPT (U/L)	34.79 ± 17.4	28.17 ± 14.3	22.42 ± 11.9	27.89 ± 12.7	24.38 ± 8.9	22.24 ± 8.4	18.00 ± 7.5	22.50 ± 11.0
γ-GT (U/L)	36.29 ± 13.6	37.08 ± 16.7	38.42 ± 18.9	36.74 ± 21.2	26.05 ± 12.7	24.91 ± 14.2	24.89 ± 14.3	24.00 ± 15.4

Values are expressed as means ± SD. BMI: body mass index; SBP: systolic blood pressure; DBP: diastolic blood Pressure; HOMA-IR: Homeostasis assessment model for insulin resistance; LDL: low-density lipoprotein; HDL: high-density lipoprotein; GOT: aspartate aminotransferase; GPT: alanine aminotransferase; γ-GT: gamma glutamyltransferase.

**Table 2:** Inflammatory biomarkers.

	Group 1				Group 2			
	Placebo		Probiotic		Probiotic		Placebo	
	t1	t2	t3	t5	t1	t2	t3	t5
CRP (mg/dL)	5.13 ± 3.7	5.78 ± 4.8	6.97 ± 7.4	5.52 ± 4.4	5.88 ± 4.4	6.12 ± 6.3	3.66 ± 2.7	4.24 ± 3.8
IL-6 (pg/mL)	2.91 ± 1.8	3.33 ± 2.4	3.12 ± 1.9	2.62 ± 2.0	2.07 ± 1.2	1.79 ± 1.2	1.37 ± 0.8	1.72 ± 1.0
IL-8 (pg/mL)	2.86 ± 1.7	2.80 ± 1.2	4.11 ± 7.1	4.23 ± 9.4	2.66 ± 1.1	2.73 ± 1.2	2.27 ± 1.0	2.28 ± 1.1
TNF-α (pg/mL)	4.70 ± 2.5	4.91 ± 2.6	4.59 ± 2.1	3.51 ± 1.7	4.05 ± 1.9	4.15 ± 2.1	3.05 ± 1.2	3.28 ± 2.0
Adiponectin (mg/L)	6.55 ± 5.1	5.95 ± 4.7	5.82 ± 3.8	6.56 ± 3.4	5.69 ± 3.7	6.91 ± 6.5	7.20 ± 4.4	8.26 ± 6.1
tPAI1 (µg/L)	9.24 ± 4.9	9.55 ± 4.3	10.55 ± 4.8	11.56 ± 7.1	9.31 ± 3.2	8.36 ± 2.6	9.08 ± 3.2	9.51 ± 3.6
P-selectin (ng/mL)	46.78 ± 19.7	46.70 ± 21.2	49.26 ± 22.8	63.47 ± 38.3	48.06 ± 16.7	40.51 ± 11.4	60.73 ± 35.4	58.09 ± 21.6
Resistin (µg/L)	17.71 ± 8.1	17.70 ± 13.7	16.99 ± 5.2	17.45 ± 10.4	15.33 ± 7.2	11.89 ± 5.2	11.08 ± 4.0	12.60 ± 5.2
HGF (pg/mL)	161.12 ± 97.9	155.73 ± 93.6	131.45 ± 65.9	162.45 ± 88.3	175.06 ± 75.1	170.17 ± 72.7	160.47 ± 68.8	157.95 ± 57.5
Leptin (µg/L)	28.56 ± 14.8	24.07 ± 12.3	23.71 ± 13.8	18.42 ± 10.5	21.97 ± 11.8	17.68 ± 10.9	13.67 ± 9.3	14.24 ± 10.4
MCP-1 (pg/mL)	107.53 ± 39.3	106.57 ± 31.7	120.07 ± 60.1	118.20 ± 46.8	108.86 ± 39.3	114.61 ± 51.5	116.31 ± 43.6	112.51 ± 41.2
sICAM (ng/mL)	73.65 ± 37.2	73.71 ± 41.1	67.86 ± 35.2	65.47 ± 32.2	74.80 ± 26.9	71.50 ± 33.1	75.17 ± 40.2	73.0 ± 41.2
MPO (ng/mL)	17.69 ± 5.9	19.96 ± 10.7	20.70 ± 13.2	30.53 ± 21.2	15.56 ± 7.8	18.14 ± 13.0	17.31 ± 12.4	19.46 ± 10.2
sVCAM (ng/mL)	494.22 ± 125.1	511.04 ± 154.8	516.47 ± 149.1	507.61 ± 138.7	489.68 ± 80.9	472.72 ± 71.4	491.62 ± 99.6	527.43 ± 74.6
LPS (ng/mL)	285.81 ± 107.2	277.90 ± 116.3	312.22 ± 126.0	326.19 ± 166.0	321.82 ± 105.4	316.83 ± 124.0	309.91 ± 136.7	308.70 ± 131.4
LBP (ng/mL)	731.26 ± 512.2	782.55 ± 323.0	635.96 ± 294.2	747.39 ± 272.6	837.47 ± 423.5	742.81 ± 349.2	833.63 ± 560.5	855.78 ± 663.1

Values are expressed as means ± SD. CRP: C reactive protein; IL: interleukin; TNF-α: tumor necrosis factor alpha; tPAI1: plasminogen activator inhibitor-1; HGF: hepatocyte growth factor; MCP-1: monocyte chemoattractant protein 1; sICAM: soluble intracellular adhesion molecules 1; sVCAM: soluble vascular cell adhesion molecule 1; MPO: myeloperoxidase; LPS: lipopolysaccharide; LBP: lipopolysaccharide-binding protein.

## Supplementary Materials

The following are available online at https://www.mdpi.com/2072-6643/11/8/1761/s1: Figure S1, Rarefaction curves; Figure S2, Alpha diversity measured by means of the Shannon index (H); Figure S3, Bacterial beta diversity; Table S1, Gastrointestinal microbiome normalized data.

## References

[1] Engin A. (2017). The Definition and Prevalence of Obesity and Metabolic Syndrome. Results Probl. Cell Differ..

[2] Fasshauer M., Blüher M. (2015). Adipokines in health and disease. Trends Pharmacol. Sci..

[3] Bäckhed F., Ding H., Wang T., Hooper L.V., Koh G.Y., Nagy A., Semenkovich C.F., Gordon J.I. (2004). The gut microbiota as an environmental factor that regulates fat storage. Proc. Natl. Acad. Sci. USA.

[4] Bäckhed F., Manchester J.K., Semenkovich C.F., Gordon J.I. (2007). Mechanisms underlying the resistance to diet-induced obesity in germ-free mice. Proc. Natl. Acad. Sci. USA.

[5] Turnbaugh P.J., Ley R.E., Mahowald M.A., Magrini V., Mardis E.R., Gordon J.I. (2006). An obesity-associated gut microbiome with increased capacity for energy harvest. Nature.

[6] Zupancic M.L., Cantarel B.L., Liu Z., Drabek E.F., Ryan K.A., Cirimotich S., Jones C., Knight R., Walters W.A., Knights D. (2012). Analysis of the gut microbiota in the older order Amish and its relation to the metabolic syndrome. PLoS ONE.

[7] Turnbaugh P.J., Hamady M., Yatsunenko T., Cantarel B.L., Duncan A., Ley R.E., Sogin M.L., Jones W.J., Roe B.A., Affourtit J.P. (2009). A core gut microbiome in obese and lean twins. Nature.

[8] Le Chatelier E., Nielsen T., Qin J., Prifti E., Hildebrand F., Falony G., Almeida M., Arumugam M., Batto J.M., MetaHIT Consortium (2013). Richness of human gut microbiome correlates with metabolic markers. Nature.

[9] Cani P.D., Bibiloni R., Knauf C., Waget A., Neyrinck A.M., Delzenne N.M., Burcelin R. (2008). Changes in gut microbiota control metabolic endotoxemia-induced inflammation inhigh-fat diet induced obesity and diabetes in mice. Diabetes.

[10] Sanders M.E. (2008). Probiotics: Definition, sources, selection, and uses. Clin. Infact. Dis..

[11] Karczewski J., Troost F.J., Konings I., Dekker J., Kleerebezem M., Brummer R.-J.M., Wells J.M. (2010). Regulation of human epithelial tight junction proteins by Lactobacillus plantarum in vivo and protective effects on the epithelial barrier. Am. J. Physiol. Liver Physiol..

[12] Miglioranza Scavuzzi B., Miglioranza L.H., Henrique F.C., Pitelli Paroschi T., Lozovoy M.A., Simao A.N., Dichi I. (2015). The role of probiotics on each component of the metabolic syndrome and other cardiovascular risks. Expert Opin. Ther. Targets.

[13] Ren C., Zhang Q., De Haan B.J., Zhang H., Faas M.M., De Vos P. (2016). Identification of TLR2/TLR6 signalling lactic acid bacteria for supporting immune regulation. Sci. Rep..

[14] Bernini L.J., Simão A.N., Alfieri D.F., Lozovoy M.A., Mari N.L., de Souza C.H., Dichi I., Costa G.N. (2016). Beneficial effects of Bifidobacterium lactis on lipid profile and cytokines in patients with metabolic syndrome: A randomized trial. Effects of probiotics on metabolic syndrome. Nutrition.

[15] Barreto F.M., Simão A.N., Morimoto H.K., Lozovoy M.A., Dichi I., Miglioranza H.D.S. (2014). Beneficial effects of Lactobacillus plantarum on glycemia and homocysteine levels in postmenopausal women with metabolic syndrome. Nutrition.

[16] Sañudo Otero A.I., Criado García R., Rodríguez Nogales A., Garach Domech A., Olivares Martín M., Gálvez Peralta J.J., De La Escalera Hueso S., Duarte Pérez J.M., Zarzuelo Zurita A., Bañuelos Hortigüela O. (2016). Probiotic Strains Having Cholesterol Absorbing Capacity, Methods and Uses Thereof. Google Patents.

[17] Tenorio-Jiménez C., Martínez-Ramírez M.J., Tercero-Lozano M., Arraiza-Irigoyen C., Del Castillo-Codes I., Olza J., Plaza-Díaz J., Fontana L., Migueles J.H., Olivares M. (2018). Evaluation of the effect of Lactobacillus reuteri V3401 on biomarkers of inflammation, cardiovascular risk and liver steatosis in obese adults with metabolic syndrome: A randomized clinical trial (PROSIR). BMC Complement. Altern. Med..

[18] Klindworth A., Pruesse E., Schweer T., Peplies J., Quast C., Horn M., Glöckner F.O. (2013). Evaluation of general 16S ribosomal RNA gene PCR primers for classical and next-generation sequencing-based diversity studies. Nucleic Acids Res..

[19] Bolyen E., Rideout J.R., Dillon M.R., Bokulich N.A., Abnet C., Al-Ghalith G.A., Alexander H., Alm E.J., Arumugam M., Asnicar F. (2018). QUIIME 2: Reproducible, interactive, scalable, and extensible microbiome data science. Peer J. Prepr..

[20] Arteaga F., Ferrer F. (2002). Dealing with missing data in MSPC: Several methods, different interpretations, some examples. J. Chemom..

[21] Wellek S., Blettner M. (2012). On the proper use of the crossover design in clinical trials: Part 18 of a series on evaluation of scientific publications. Dtsch. Arztebl. Int..

[22] Bibby J., Kent J., Mardia K. (1979). Multivariate Analysis.

[23] Barker M., Rayens W. (2003). Partial least squares for discrimination. J. Chemom..

[24] Smilde A.K., Jansen J.J., Hoefsloot H.C., Lamers R.J., Van Der Greef J., Timmerman M.E. (2005). ANOVA-simultaneous component analysis (ASCA): A new tool for analyzing designed metabolomics data. Bioinformatics.

[25] Bokulich N.A., Dillon M.R., Zhang Y., Rideout J.R., Bolyen E., Li H., Albert P.S., Caporaso J.G. (2018). q2-longitudinal: Longitudinal and Paired-Sample Analyses of Microbiome Data. mSystems.

[26] Tripolt N., Leber B., Blattl D., Eder M., Wonisch W., Scharnagl H., Stojakovic T., Obermayer-Pietsch B., Wascher T., Pieber T. (2013). Short communication: Effect of supplementation with Lactobacillus casei Shirota on insulin sensitivity, β-cell function, and markers of endothelial function and inflammation in subjects with metabolic syndrome—A pilot study. J. Dairy Sci..

[27] Jaganathan R., Ravindran R., Dhanasekaran S. (2018). Emerging Role of Adipocytokines in Type 2 Diabetes as Mediators of Insulin Resistance and Cardiovascular Disease. Can. J. Diabetes.

[28] Weigert C., Hennige A.M., Lehmann R., Brodbeck K., Baumgartner F., Schaüble M., Häring H.U., Schleicher E.D. (2006). Direct Cross-talk of Interleukin-6 and Insulin Signal Transduction via Insulin Receptor Substrate-1 in Skeletal Muscle Cells. J. Biol. Chem..

[29] Glowinska B., Urban M., Peczynska J., Florys B., Głowińska-Olszewska B. (2005). Soluble adhesion molecules (sICAM-1, sVCAM-1) and selectins (sE selectin, sP selectin, sL selectin) levels in children and adolescents with obesity, hypertension, and diabetes. Metabolism.

[30] Guzmán-Guzmán I.P., Zaragoza-García O., Vences-Valázquez A., Castro-Alarcón N., Muñoz-Valle J.F., Parra-Rojas I. (2016). Circulating levels of MCP-1, VEGF-A, sICAM-1, sVCAM-1, sE-selectin and sVE-cadherin: Relationship with components of metabolic síndrome in young population. Med. Clin. (Bar).

[31] Dao M.C., Everard A., Aron-Wisnewsky J., Sokolovska N., Prifti E., Verger E.O., Kayser B.D., Levenez F., Chilloux J., Hoyles L. (2016). Akkermansia muciniphila and improved metabolic health during a dietary intervention in obesity: Relationship with gut microbiome richness and ecology. Gut.

[32] Everard A., Belzer C., Geurts L., Ouwerkerk J.P., Druart C., Bindels L.B., Guiot Y., Derrien M., Muccioli G.G., Delzenne N.M. (2013). Cross-talk between Akkermansia muciniphila and intestinal epithelium controls diet-induced obesity. Proc. Natl. Acad. Sci. USA.

[33] de Vos W.M. (2017). Microbe profile: Akkermansia muciniphila: A conserved intestinal symbiont that acts as the gatekeeper of our mucosa. Microbiology.

[34] Zhang X., Shen D., Fang Z., Jie Z., Qiu X., Zhang C., Chen Y., Ji L. (2013). Human gut microbiota changes reveal the progression of glucose intolerance. PLoS ONE.

[35] Qin J., Li Y., Cai Z., Li S., Zhu J., Zhang F., Liang S., Zhang W., Guan Y., Shen D. (2012). A metagenome-wide association study of gut microbiota in type 2 diabetes. Nature.

[36] Stadlbauer V., Leber B., Lemesch S., Trajanoski S., Bashir M., Horvath A., Tawdrous M., Stojakovic T., Fauler G., Fickert P. (2015). Lactobacillus casei Shirota supplementation does not restore gut microbiota composition and gut barrier in metabolic syndrome: A randomized pilot study. PLoS ONE.

