# Supplementary material for: Lactobacillus reuteri V3401 Reduces Inflammatory Biomarkers and Modifies the Gastrointestinal Microbiome in Adults with Metabolic Syndrome: The PROSIR Study
Source: Nutrients. 2019 Jul 31;11(8):1761. doi: 10.3390/nu11081761 (PMC6723328; doi:10.3390/nu11081761)
Supplement: Supplementary file 1 [file nutrients-11-01761-s001.zip › supplementary figures-547567.pdf]

Figure S1. Rarefaction curves

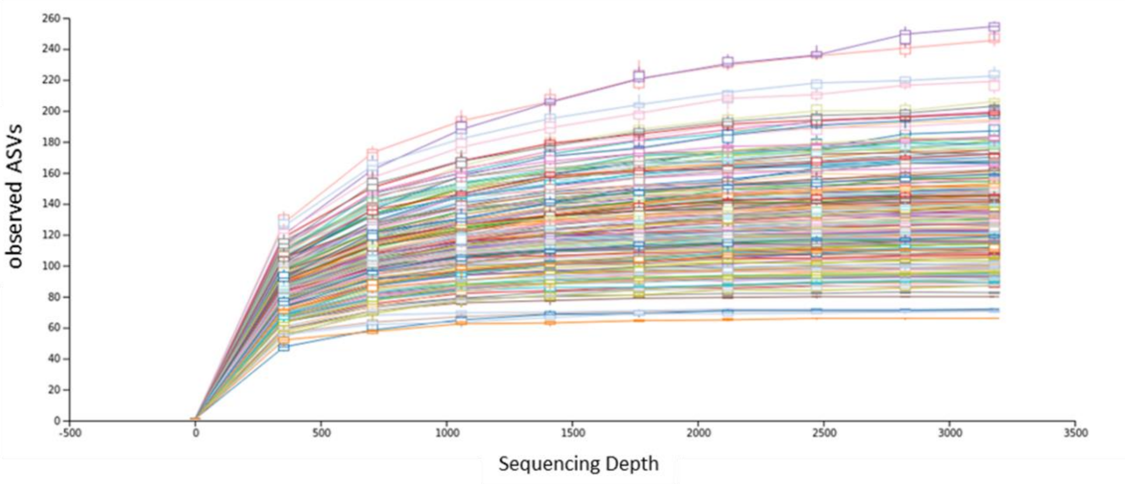

Figure S2. Alpha diversity measured by means of Shannon Index (H)

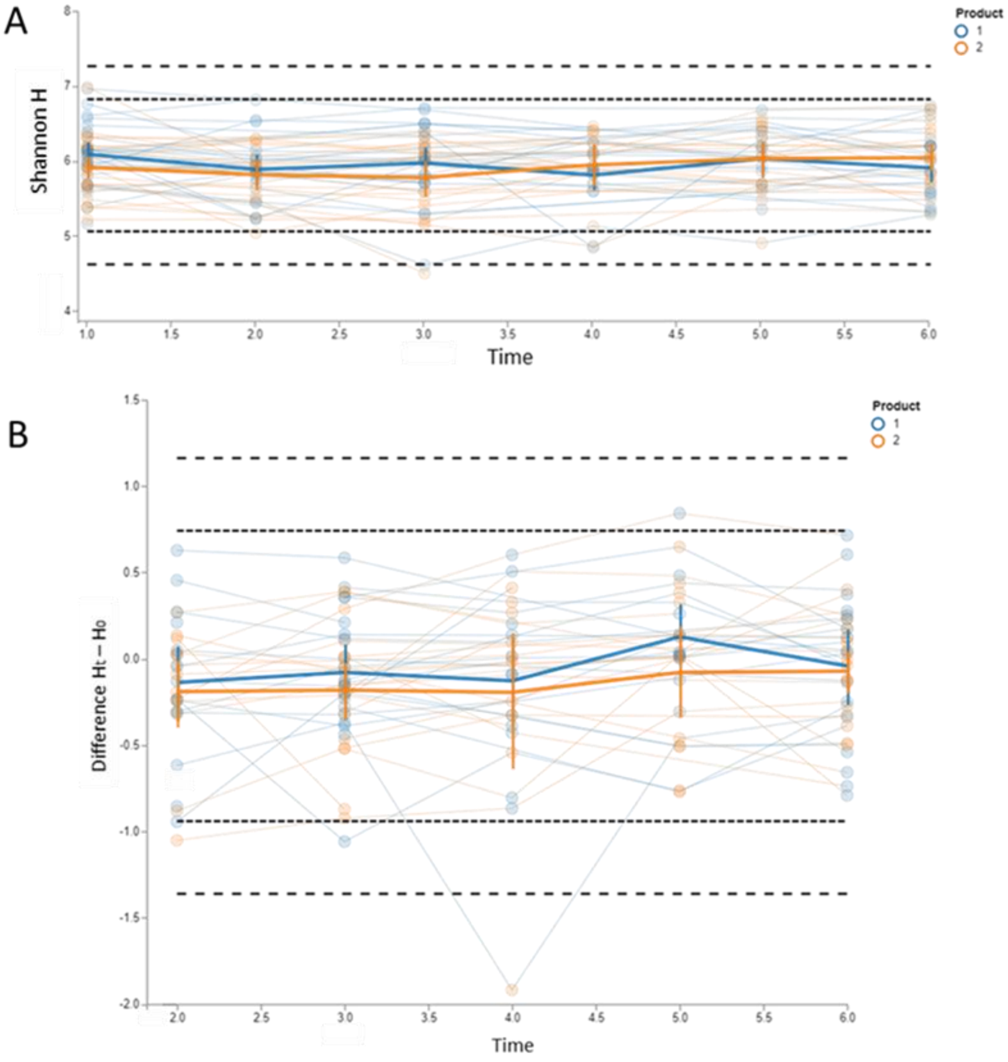

Figure S2. Shannon Index. The figure represents the alpha diversity across the study. The thick lines with error bars represent the means ± standard desviation of both group (Group 1: blue lines; group 2: orange lines). Dashed lines represent the means ± 2 and 3 times standard desviations. Group 1 started the intervention receiving the placebo (t1, t2 and t3) and then was swichted to receive the probiotic strain (t4, t5 and t6). Group 2 started the intervention receiving the probiotic strain (t1, t2 and t3) and then was swicthed to receive the placebo (t4, t5 and t6). A: Temporal trend of Shannon index (H). B: Temporal trend of the Shannon index (H) between treatment and baseline (delta) (Ht-Ho).

Figure S3. Bacterial beta diversity

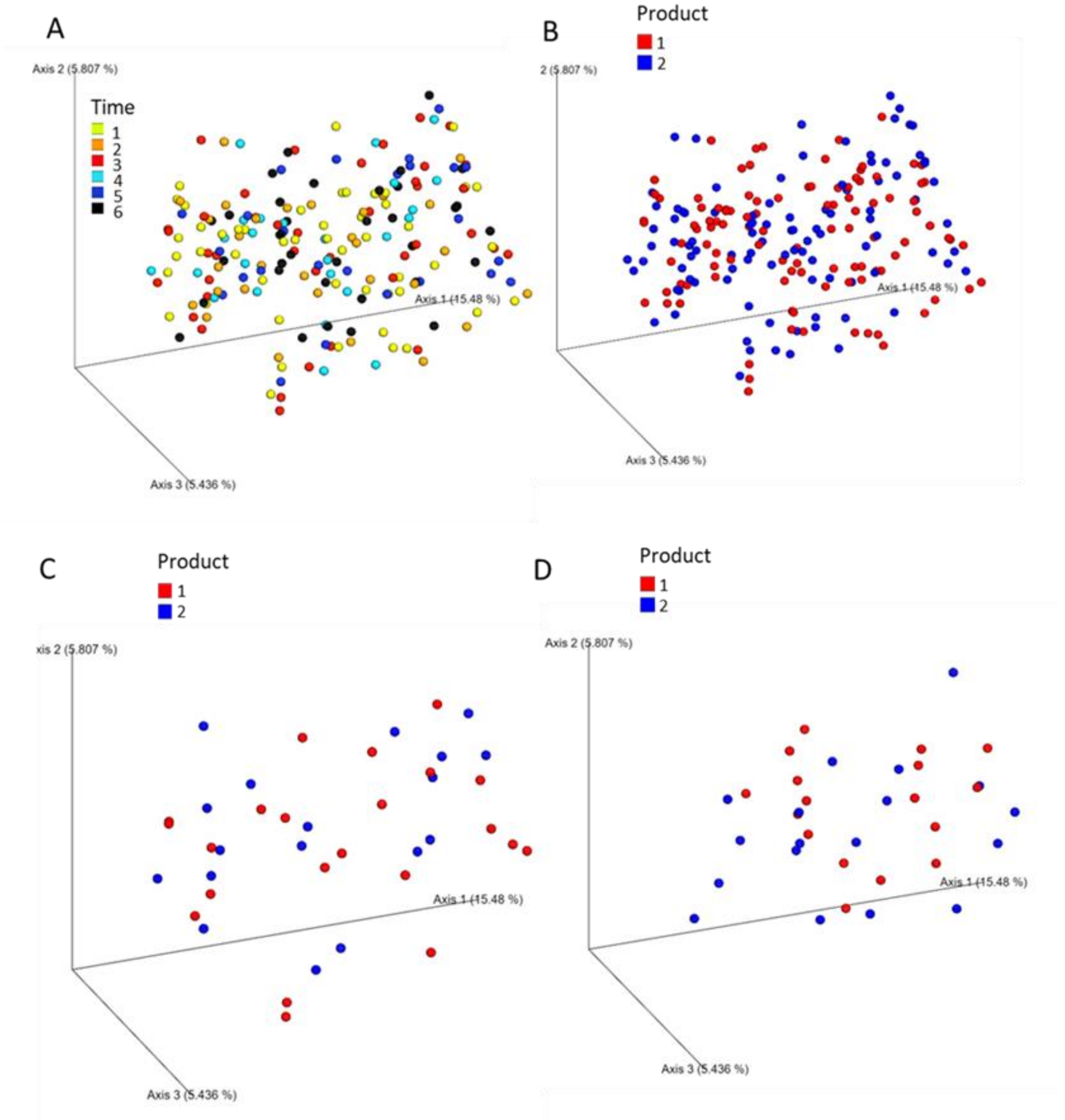

Figure S3. Principal Coordinates analysis (PCoA). Unweighted Unifrac analysis and distances estimated among samples. The three-dimensional scatter plot showed the distances of microbial communites among participants by time (First intervention t1, t2 and t3; second intervention t4, t5 and t6) (A), by treatment (B), and the distances among patients at the end of the first intervention (t3) (C) and at the end of the second intervention (t6) (D).
